# The Role of Antiviral Therapy for HBV-Related Hepatocellular Carcinoma

**DOI:** 10.4061/2011/416459

**Published:** 2011-05-30

**Authors:** Liang-He Yu, Nan Li, Shu-Qun Cheng

**Affiliations:** ^1^Eastern Hepatobiliary Surgery Hospital, The Second Military Medical University, Shanghai 200438, China; ^2^Tumor Comprehensive Treatment Department, Eastern Hepatobiliary Surgery Hospital, The Second Military Medical University, 225 Changhai Road, Shanghai 200438, China

## Abstract

Hepatocellular carcinoma (HCC) is a highly prevalent and lethal cancer worldwide; despite the curative treatment for HCC, the rate of tumor recurrence after hepatectomy remains high. Tumor recurrence can occur early (<2 years) or late (>2 years) as metastases or de novo tumors. Several tumor factors were associated with HCC recurrence; high hepatitis B virus (HBV) load is the major risk factor for late recurrence of HCC after resection. Preoperative antiviral therapy improves liver function, and postoperative reduce HCC recurrence. In this paper, we focus on antiviral treatment to improve the liver function, prevent recurrence, and lengthen the overall survival for HBV-related HCC.

## 1. Introduction

Hepatocellular carcinoma (HCC) is one of the major health problems worldwide, ranking as the third leading cause of cancer-related mortality in the world, and the second in China [1]. The annual incidence of HCC in hepatitis B cirrhotic patients can run as high as 3–5%, and one-third will develop HCC in their lifetime [2]. For patients with hepatitis B virus-related HCC (HBV-related HCC), early-stage tumors and preserved hepatic function, liver resection, and liver transplantation offer the best therapeutic choice. The palliative treatment modalities include transarterial chemoembolization (TACE) and targeted systemic chemotherapy with sorafenib. Unfortunately, despite the continuing efforts for the curative treatment HCC with surgical resection, the rate of tumor recurrence after hepatectomy remains high (>70% at 5 years), which still limits survival of the patients [3]. Several factors are reported to be associated with an increased risk of HCC recurrence after surgical resection, including tumor characteristics such as multiplicity, size, and portal invasion, AFP level, PIVKA-II level, and hepatic functional parameters such as albumin level, PT, and Child-Pugh class [4, 5]. Recently, accumulating evidence has shown that a high serum hepatitis B viral (HBV) DNA level is another risk factor for de novo HCC development in HBV carriers irrespective of hepatitis activity [6, 7]. Additionally, some investigators have shown the viral replicative status of subjects as a predictor of postoperative recurrence of HCC [8, 9]. Therefore, it was very significant and interesting to investigate of the molecular mechanism of the direct carcinogenic effect of HBV, and it may help us to clarify additional therapeutic targets for HCC prevention. However, in previous studies, the relation between HBV load and the recurrence of HCC after resection may be confounded by other major risk factors for recurrence, such as macroscopic vascular invasion or noncurative resection. In this paper we review the incidence of HBV-related HCC and its impact on the prevention of recurrence with antivirus therapy. 

## 2. The Incidence and Surveillance of HBV-Related HCC

Individuals with chronic hepatitis B (CHB) infection have a risk of developing HCC, that is, 100-fold greater than the persons who are not infected [10]. Most carriers of CHB, including Asians, Africans, and a proportion of persons in Mediterranean countries, acquire the infection at birth or within the first 1 to 2 years after birth [11]. Once chronic infection is established, complete eradication of the virus is still not possible, and these patients are facing the risk of HCC development [10]. Previous longitudinal studies have shown that genotype B patients have an earlier and more frequent hepatitis B e antigen (HBeAg) seroconversion than genotype C patients, [12, 13] indicating that genotype C patients may have more severe liver disease than genotype B patients. In addition, genotype C HBV was associated with increased viral load, and associations of HBV genotype and viral load with HCC risk were additive. This suggests that viral load and genotype determination may be important factors to consider regarding screening program for the detection of HCC and treatment indication. 

In patients with CHB, screening for HCC is necessary even after clearance of serum hepatitis B surface antigen (HBsAg) and HBV DNA and remission of hepatitis, especially in those with a high antihepatitis B core antibody (anti-HBc) titer [14, 15] because the oncogenic potential due to occult HBV infection or the integration of HBV DNA is considered to continue [16]. The earlier the seroconversion of HBeAg, the better the clinical outcome of HBV carriers. A single randomized study from China comparing surveillance and nonsurveillance in HBV patients using periodic serum AFP and abdominal ultrasound at 6-month intervals demonstrated the benefit of surveillance in terms of reduced mortality [17]. With AFP assays and the development of modern imaging systems, such as ultrasonography (US), computed tomography (CT), and magnetic resonance imaging (MRI), more and more hepatitis B-related HCCs can be detected and diagnosed and hepatectomy early. However, the prognosis of HCC remains unsatisfactory, even after curative resection, yet with recurrence of HBV-related HCC is extremely high [18], which is also the main cause of death, in addition to concomitant hepatic decompensation. It has shown that with the successful implementation of HCC surveillance and curative treatment, more patients could avoid the risk of early recurrence and thus survive longer. 

## 3. The Mechanisms of HBV-Related HCC Recurrence

It is well known that there are two distinct types of HCC recurrence: tumors grown from dissemination of the primary tumor and de novo tumors arising from the “field effect” in diseased liver [19, 20]. That is, the latter is clonally independent from the primary tumor. However, the mechanism for recurrent carcinogenesis associated with HBV in the remaining liver in patients who have undergone curative resection remains unclear. Over the past years, studies have suggested that high viral load is via direct and indirect ways which are thought to be involved for recurrence [21]. It is possible that sustained viremia and subsequent active viral replication may contribute to the carcinogenic process. Active replication of HBV may initiate malignant transformation through a direct carcinogenic mechanism by increasing the probability of viral DNA insertion in or near proto-oncogenes, tumor-suppressor genes, or regulatory elements of cellular DNA [22, 23]. The integration of viral DNA may increase the production of transactivator protein hepatitis B X antigen, which may promote the neoplasia of hepatocytes, as well as, bind to the p53 tumor-suppressor gene and disrupt its functions [24, 25]. Indirectly, continuing HBV replication can also induce chronic liver fibrosis and inflammation and mediate alteration in transforming growth factor-beta1 (TGF-*β*1) and alpha-M production, thereby leading to carcinogenesis [26, 27]. 

Imamura proposed a convenient framework to clinically differentiate each type of recurrence as “early” or “late” recurrence based on a cutoff of 2 years after surgery [28]. This framework has made it possible to assess risk factors for each type of recurrence [29]. For one thing, early recurrence, which appears within 2 years after surgery, is associated with tumor-related factors including the presence of vascular invasion and additional tumor sites besides the primary lesion (satellite lesion), which is consistent with the notion that this type of recurrence is tumor dissemination as a consequence of malignant characteristic of the primary tumor. For another, late recurrence, which appears more than 2 years after surgery, is considered to be associated with the severity of hepatic inflammation and liver damage closely linked to the “field effect.” Early intrahepatic recurrence has poorer prognosis than late intrahepatic recurrence. Discrimination of these types of recurrence is clinically important because the biological basis producing each recurrence is different, and the following therapeutic intervention should be considered accordingly [30]. Recently, Kim et al. reported that persistent viremia is associated with disease-free survival after 12 months of surgery, suggesting its association with late recurrence [31]. And Wu et al. also evaluated clinical variables together with HBV-related factors including viral load, genotype, and recurrent mutations, for their prognostic implication with respect to early and late recurrence in 193 HBV-related HCC patients [9]. During the median followup of 5 years, 134 patients (69%) had HCC recurrence [19]. It was found that tumor-related factors: microvascular invasion, positive cut margin, and high serum AFP level were associated with the risk of early recurrence, whereas liver inflammation/damage-related factors: histological inflammation and ICG-15 retention rate were independently associated with the risk of late recurrence [9]. Interestingly, the high HBV viral load was found to be associated with the risk of late, but not early recurrence, probably because the high HBV DNA level is the most functional measure reflecting the exposure to the direct carcinogenic effect of HBV. 

## 4. The Necessity of Antiviral Treatment on HBV-Related HCC

As above mentioned, for patients with CHB, serum HBV DNA levels have emerged as the key risk factor for the development of HCC. This may argue for an earlier antiviral intervention, before the development of cirrhosis, to prevent HCC development, and even more, as adjuvant therapy after the resection HCC for the patients with a high HBV DNA level to prevent late recurrence. But only a few recent studies have evaluated HBV replication status as a predictor of HCC recurrence [8, 32], and the interpretation of their results was complicated by the use of antiviral therapy. Notably, several studies found a significant association between high HBV load and increased risk of HCC and liver cirrhosis [6, 33]. Elevations in serum HBV DNA level are not only a major risk factor for HCC recurrence, but also the risk factor most amenable to modification.

Several large cohort studies from China, Taiwan, and Senegal reported that high serum HBV DNA levels at the time of enrollment were associated with an increased risk of cirrhosis and HCC. Therefore, it was suggested that serum HBV DNA level, and not only liver disease activity, might be used as an indication for antiviral therapy. In a large prospective study of 3653 HBV carriers in Taiwan, Chen and colleagues [6] reported that 164 had HCC after a mean follow-up of 11.4 years. The incidence of HCC correlated with serum HBV DNA level at entry in a dose-response relationship. The authors concluded that high serum HBV DNA levels (>10^4^ copies/mL) were a strong predictor of HCC independent of HBeAg, ALT, and the presence of cirrhosis. Moreover, a subanalysis showed that spontaneous decline of viremia levels from levels higher than 10^5^copies/mL to levels below 10^4^ copies/mL was associated with a reduced risk of HCC development by comparison with patients who maintained high viremia levels. Thus, the authors emphasize that effective control of HBV replication with antiviral therapy may lower the risk of HCC. 

### 4.1. The Impact of Antiviral Therapy on HBV Load for HBV-Related HCC

Antiviral treatment may render patients with HBV-related HCC better able to tolerate HCC treatments and may improve prognosis. However, the efficacy of antiviral therapy on HBV viral status and underlying liver function in patients is still unclear. Many questions remain to be answered in terms of clinical management of CHB to improve the prevention of HCC late recurrence: (1) can the correlations between high viral load and HCC recurrence risk be generalized to all HBV carriers whatever their HBeAg status, alanine aminotransferase (ALT) levels, and stage of CHB? and (2) the major clinical question is whether antiviral therapy can prevent HCC late recurrence. Lin conducted a study in order to evaluate the effectiveness of IFN-*α* with 16 HBV patients after medical ablation therapy for primary tumors [34]. They found that HCC recurred in four of four (100%) untreated patients and in four of 12 (33.3%) IFN-*α* treated patients (*P* = .0384). They concluded that IFN-*α* therapy may reduce HCC recurrence after medical ablation for primary HCC although the sample size was too small to reach a firm conclusion. The Asian Cirrhosis Lamivudine multicenter randomized controlled trial (RCT) study showed that lamivudine can reduce disease progression in HBV-related cirrhosis, including an approximately 50% decrease in HCC incidence. Such efficacy was achieved despite the emergence of drug resistance in approximately 50% of cases. In a small study, Hung has reported 72 patients who underwent HCC resection and found that patients with viral load of more than 2000 IU/mL had a significantly higher risk of HCC recurrence after resection [8], with viral load being the most important correctable risk factor for HCC recurrence (odds ratio 22.3, 95% CI 3.3–151, *P* = .001). However, only 10 patients were treated with lamivudine, and none of these patients had HCC recurrence compared to those without antiviral therapy, but this finding could be due to the small sample size. Recently, a nonrandomized comparative study for postoperative antiviral treatment was conducted on patients who underwent curative hepatectomy for advanced HCC [35]. Patients in the treatment group (*n* = 43) received lamivudine with or without adefovir dipivoxil, while the control group (*n* = 36) received no antiviral treatment. The treatment group had a significantly higher HBeAg seroconversion rate (57.2% versus 5.6%; *P* < .05). And HBV DNA suppression rates at 12 and 24 months were 87.2% and 98.0%, respectively, in the treatment group, compared with 2.8% and 3.6%, respectively, in the control group (*P* < .05). The data of this study has shown the efficacy of postoperative antiviral therapy in suppressing viral replication. Thus, to improve the liver function, antiviral therapy should be initiated in patients with detectable serum HBV DNA level after resection. Further RCTs with larger numbers of patients and longer follow-up periods are urgently necessary to clarify whether the efficacy of antiviral therapy on HBV load for HBV-related HCCs' patients who underwent hepatectomy. 

### 4.2. The Impact of Antiviral Therapy on Perioperative Liver Function for HBV-Related HCC

Recent reports found that an effective preoperative anti-HBV therapy could contribute to improve liver function. Thia reported the incidence of postoperative HBV and exacerbation of chronic hepatitis B (ECHB) [36], that transient elevation of serum ALT in the first week after resection occurred in 92% of cases and resolved by the second week. The peak serum ALT was 222.0 IU/L and declined by week 2 after resection. The serum activities of AST and ALT were significantly higher in the patients with a high viral load than in those with a low viral load (*P* = .0005 and *P* = .0089, resp.) [37]. Notably, the AST and ALT activities were significantly higher in the patients with a high viral load, and the percentage of patients with moderately or severe active hepatitis was significantly higher in the patients with a high viral load. A previous study indicated that reoperative prealbumin was 223.7 ± 56.0 mg/L versus 226.1 ± 60.5 mg/L (*P* = .859), albumin/ globulin ratio was 1.4 ± 0.2 versus 1.3 ± 0.2 (*P* = .129), and *γ*-globulin on protein electrophoresis was 20.0 ± 4.3 versus 20.6 ± 4.4, in the treatment and the control groups respectively (*P* = .540) [35]. There were no significant differences between the two groups, and at the sixth postoperative month of followup, prealbumin was 201.3 ± 52.4 mg/L versus 224.3 ± 85.8 mg/L (*P* = .148), and albumin/globulin ratio was 1.3 ± 0.3 versus 1.2 ± 0.3 (*P* = .114). By analysis, there was no correlation between prealbumin and logarithmic difference in HBV DNA level (*P* = .688), and between albumin/globulin ratio and logarithmic difference in HBV DNA level (*P* = .130). However, the treatment group had a significantly greater increase in residual liver volume per unit surface area following hepatectomy (78.0 ± 40.1 cm^3^/m^2^ versus 35.8 ± 56.0 cm^3^/m^2^) at the sixth postoperative month. these results indicated that remnant liver functions in the nucleotide analog group were maintained better than those in the control group. Remnant liver function is an important factor in selecting further treatment for HCC recurrence and is a prognostic factor for the survival rate. Therefore, patients treated by nucleotide analogs have more aggressive therapies for HCC recurrence, resulting in improving the cumulative survival rate. 

### 4.3. The Impact of Antiviral Therapy on Postoperative Recurrences for HBV-Related HCC

As we all known, prolonged suppression of HBV replication with nucleoside or nucleotide analogs may reduce the risk of HBV-related HCC development [38]. It aslo may prevent postsugery HCC recurrence. In a cohort study design, Kim et al. [31] have reported patients on antiviral therapy at the time of liver resection. 157 patients were included, among them 89 were non-viremic and 68 were viremic. the 5-year cumulative recurrence rate was 73% for viremic group compared to the non-viremic group by 55% (*P* = .043). However, in the previous study [35] we found there was no significant difference in recurrence rate after surgery in the two groups (76.7% versus 91.7%; *P* = .077), after a median follow-up of 12 months. The median time to recurrence in the treatment and control groups were 7.0 and 6.0 months, respectively (*P* = .072). Indeed, Kuzuya et al. [39] study has also reported that the cumulative recurrence rates of HCC after initial and curative treatment for HCC did not decrease by the administration of nucleotide analog. Consequently, to confirm the efficacy of nucleotide analogs against the recurrence of HCC, further studies with a larger number of patients and longer follow-up period are needed to address this question. 

### 4.4. The Antiviral Therapy on the Overall Survial for HBV-Related HCC

Up till now, there have been very few studies that have documented whether antiviral therapy is beneficial to the survival after treatment for HCC. In theory, modulation in liver function may not only affect survival directly but also indirectly by influencing the patient's tolerance to various treatments for recurrence. Miao reported in a meta-analysis study that [40], postoperative antiviral therapy as a whole has been shown to reduce HCC recurrence at year 1, 2, 3, and 5. Several small sample size of RCT and NRCTs have evaluated the efficacy and outcome of antiviral therapies in patients with HBV-related HCC after curative treatment, however, clinically meaningful differences are less. In the Kuzuya et al. study [39], no significant differences regarding the recurrence rates of HCC was found (*P* = .622). the cumulative HCC recurrence rates at 1, 2, and 3 years in the lamivudine group were 13.5%, 35.1%, and 35.1%, respectively, while those in the control group were 13.4%, 39.2% and 53.2%, respectively. All 16 patients in the lamivudine group still alive during their follow-up period, but six of 33 patients died in the control group. There were no significant differences with regard to survival rate between the two groups; however, the survival rates in the lamivudine group tended to be higher than those in the control group (*P* = .063). 

In the RCT of Liaw et al. study, continuous treatment with lamivudine has been shown to delay clinical progression in patients with CHB and advanced fibrosis or cirrhosis by significantly reducing the incidence of hepatic decompensation and the risk of HCC [38]. HCC occurred in 3.9% in the lamivudine group and 7.4% in the placebo group (hazard ratio, 0.49; *P* = .047). The previous study also found that after a median follow-up of 12 months, 41 patients of the treatment group and 36 patients of the control group had died [35]. The 1- and 2-year overall survival rates were 41.9% and 7.0%, respectively, for the treatment group, and 33.3% and 0%, respectively, for the control group. (*P* = .0094) [35]. The 1- and 2-year disease-free survival rates were 23.3% and 2.3%, respectively, for the treatment group, and 8.3% and 0%, respectively, for the control group. (*P* = .072). The date did not show a significant effect of postoperative antiviral therapy on HCC recurrence, but it showed a significant benefit in overall survival. Although nucleoside analogs did not reduce short-term recurrence rate or progression of disease, they promoted postoperative viral clearance, increased residual liver volume, and enhanced hepatocyte regeneration in HCC patients associated with active hepatitis B, which significantly enhanced the tolerance to subsequent therapy. As a result, the overall survival was improved for those patients with postoperative antiviral therapy. We putative that if compared with other adjuvant therapies, antiviral therapy may serve as a cost-effective and favorable alternative to improve the prognosis of patients, and long-term prospective studies of antiviral therapy in chronic HBV carriers may aslo be required. 

## 5. Character of Antiviral Drugs

### 5.1. Interferon

The first treatment that had some success against CHB was interferon alpha (IFN-*α*). IFN-*α* has both antiviral and antiproliferative properties. Meta-analyses have shown that IFN-a has a beneficial effect on HBeAg loss and sustained reduction in serum HBV DNA levels [41, 42]. The antiproliferative effects of IFN include retardation of G1/S phase transition and inhibition of cell proliferation without apoptosis [43], and also induction of antiproliferative signaling through the JAK/STAT pathway [44]. However, it is not clear whether it has potential effect on HCC prevention. Only one RCT and several case-control or cohort studies have shown its benefits for preventing HCC, particularly in cirrhotic patients who responded to therapy. In patients with decompensated cirrhosis, standard or pegylated INF-*α* is usually contraindicated or causes profound intolerance, but it is still one of the choices for the operative candidates. 

### 5.2. Nucleos(t)ide Analogs

During the last decade, the rise of oral nucleos(t)ide analogs has changed the treatment landscape for CHB. Long-term lamivudine treatment can prevent complications of HBV-related liver disease as long as viral suppresion is maintained [38]. It is considered to slow the progression of severe liver disease to cirrhosis as well as to HCC [38, 45]. However, the overwhelming majority of patients relapsed after treatment cessation. It could form (as will other nucleosides with even lower rates of resistance) the backbone of maintenance combination therapies. The major disadvantage of lamivudine treatment is the high rate of resistance observed in both HBeAg and anti-HBe-positive HCC patients. The resistance usually emerges after the first 6 months with cumulative rates of 15–25% by 12 months and 60–65% by 4 years of therapy [46].

Adefovir dipivoxil and entecavir have been shown to be safe and effective for the treatment of patients with CHB that does not respond to lamivudine [47, 48]. It is effective against both wild-type and lamivudine-resistant HBV strains [46]. In the pivotal anti-HBe positive adefovir study [49], 185 patients were randomised to placebo or adefovir 10 mg daily for 48 weeks. At 48 weeks, the adefovir-treated group had significant improvement when compared with placebo improvement in liver histology (64% versus 33%), reductions in HBV DNA (3.91 versus 1.35 log copies/mL), normalisation of ALT (72% versus 29%), an undetectable HBV DNA (<400 copies/mL), and HBeAg seroconversion (12% versus 6%). Data from a recent study of 125 patients undergoing long-term adefovir treatment indicated that 0% of patients had resistance in year 1, 11% in year 3, and 28% in year 5. Interestingly, all patients who developed adefovir resistance were not receiving lamivudine and adefovir combination therapy, but adefovir monotherapy [50]. Moreover, based on in vitro studies and limited clinical data, lamivudine has been shown to be effective in patients with adefovir-resistant HBV [51, 52]. For these reasons above, we deduce that lamivudine and adefovir combination therapy may be better than adefovir monotherapy. Because of the suboptimal profile of both lamivudine and adefovir monotherapy for patients with HBV-decompensated cirrhosis, a first-line combined indefinite use of lamivudine and adefovir is recommended in several guidelines, despite the lack of data on efficacy and safety for such a strategy [53]. Tenofovir is the latest antiviral medicine and has similar safety profile as adefovir in the phase III trials. 

## 6. Future Perspectives

The goal of antiviral therapy for CHB is to prevent the development of cirrhosis and HCC. To date, several guidelines for the treatment of CHB patients are presented. However, there are no uniform guidelines globally for the usage of antivirals in the treatment CHB, let alone antivirals on HBV-related HCC at present. Currently the definite indications for the treatment of CHB are serum HBV DNA levels greater than 10^5^ copies/mL and ALT levels more than 2 × ULN [54, 55]. According to the American Association for the Study of Liver Diseases and Asian Pacific Association for the Study of the Liver guidelines, biopsy-confirmed liver disease is a key requisite for initiating treatment in patients older than 40 years of age with serum ALT levels between 1 and 2 × ULN. If cirrhosis is present, an HBV DNA level greater than 10^5^ copies/mL is the sole criterion for treatment. Treatment end points, including reduction of HBV DNA levels to less than 10^5^ copies/mL, ALT normalization, HBeAg loss, HBsAg loss, and improvement in liver histology, are used to determine treatment success. These guidelines may apply to patients who acquire the HBV infection during adolescence or adulthood but are less suitable for most HBV carriers, who are infected in early life. In light of the reports, the liver-related mortality and complications were greater in patients with ALT levels between 0.5 and 1 × ULN than in those with ALT levels less than 0.5 × ULN [56, 57], and the revision of the ULN for patients with CHB is recommended by some guidelines [11, 55, 58]. Therefore, HBeAg seroconversion may not be an adequate end point for these patients; the ideal treatment end points are permanent suppression of HBV DNA to levels undetectable by polymerase chain reaction and reduction of ALT levels to less than 0.5 × ULN. In the current treatment guidelines, antiviral treatments should be started among cirrhotic patients despite lower HBV DNA levels [59]. Treatment is based on HBV replication status and stage of liver disease, modulated by the age of the patient, HBeAg status, and patient preference. 

However, results of experimental studies suggest that early treatment intervention is necessary to prevent liver cell damage and decrease viral genome integration. Another important finding from recent studies is that viral genome integration persists despite antiviral induced viral suppression and cccDNA clearance. This was shown to be associated with the expansion of cellular clones not expressing viral antigens. Therefore, screening of HCC remains mandatory even in patients with sustained viral suppression induced by antiviral therapy to detect HCC for which curative treatments can be proposed. Late recurrence mostly corresponds to de novo carcinogenesis associated with HBV viremia as well as the “field effect” in HBV-related HCC. This may support prioritized use of anti-HBV treatment as adjuvant therapy after the resection or ablation of HCC for the patients with a high HBV DNA level to prevent late recurrence, given that the incidence rate of recurrence is higher than that of the initial HCC development [60]. Furthermore, investigation of the molecular mechanism of the direct carcinogenic effect of HBV may help clarify additional therapeutic targets in terms of HCC prevention. Currently, available evidence, mostly obtained from PCR-based assays with limited scale, has identified a handful of the genomic integrations potentially affecting the function of genes, for example, cyclin A and telomerase reverse transcriptase, in a sporadic manner [61, 62]. 

Recently, emerging genomics technology like high-throughput sequencing [63, 64] may provide a more comprehensive view of the critical recurrent oncogenic integration events. Combination of the new sequencing assay with chromatin immunoprecipitation (ChIP-Seq) [65], may help to identify oncogenic transactivation by HBV proteins to obtain complementary information of the direct carcinogenic effect caused by HBV. Although an antineoplasm effect is not expected by nucleoside analogues, the incidence rate of HCC would decrease due to a cessation of hepatitis. Future RCTs with larger sample size, longer followup, and regular HBV DNA monitoring will be needed to substantiate the beneficial effects of antiviral therapies which where performed in the light of the recently updated HBV treatment guidelines, on the prognosis of HCC and HCC recurrence. Of course, they are focusing on the need to lower the threshold of commencing antiviral treatment based on HBV-related HCC. 
